# Prevalence, awareness, treatment, control and risk factors associated with hypertension in Lebanese adults: A cross sectional study

**DOI:** 10.21542/gcsp.2018.6

**Published:** 2018-03-14

**Authors:** Bouchra Bakr Mouhtadi, Reem Mohamad Najib Kanaan, Mohammad Iskandarani, Mohamad Khaled Rahal, Dalal Hammoudi Halat

**Affiliations:** 1 Lebanese International University, Beirut, Lebanon; 2 Lebanese International University, Beirut and Bekaa, Lebanon

## Abstract

**Background:** The prevention and control of hypertension is an essential component for reducing cardiovascular disease burden. Hypertension is an important public health issue, yet few studies have examined its current status among the Lebanese population.

**Objective:** To examine the prevalence, awareness, treatment and control of hypertension and its associated risk factors among Lebanese adults.

**Methods:** A cross-sectional study was conducted between December 2014, and May 2015, on adults from the five districts of Lebanon. Multistage sampling was used to enroll participants. Hypertension was defined as an average of two blood pressure (BP) measurements with systolic/diastolic blood pressure of at least 140/90 mm Hg, using an automated digital device, or the use of antihypertensive medication. A questionnaire was used to assess hypertension risk factors, awareness, treatment and control.

**Results:** Of the 1362 Lebanese adults interviewed, 399 (29.3%) had hypertension. Of these, 106 (26.5%) were aware of their condition. Sixty-nine patients (65%) of those aware, were receiving treatment, and 38 (55%) participants from those treated were controlled. The significant risk factors were sex, gender, age, family history of hypertension, obesity, and a low level of education.

**Conclusions:** Hypertension is prevalent among the Lebanese adult population and is multifactorial, but remains incompletely recognized, leading to insufficient control. Hypertension was highly prevalent in males in the age category 18-29 years. These findings show that improvements in detection, treatment, and control of hypertension among Lebanese adults, is much needed.

## Introduction

Around the globe, hypertension is the most important preventable cause of morbidity and mortality^1^. The consequences of hypertension are deleterious. It is associated with serious complications such as: stroke, intracerebral hemorrhage, heart failure, chronic kidney disease, and others. Globally, about 7.5 million deaths occur each year due to high blood pressure, this accounts for almost 12.8% of all deaths^1^. Projections show that by 2030, prevalence of hypertension is expected to increase by 7.2% from 2013 estimates^2^. The overall estimated prevalence of hypertension in the Middle East is 29.5%^3^.

In Lebanon, cardiovascular diseases are the leading cause of death. Few studies have addressed the prevalence, awareness, treatment, control and risk factors associated with hypertension. From 2005 to the beginning of 2015, no survey had been conducted to address these issues. A recent study published in 2015 showed a prevalence of 36.9% and an awareness of 53%^4^. Given that, and for updating and increasing dependability of data regarding hypertension in Lebanon, this study was implemented to assess prevalence, awareness, treatment, control and risk factors associated with hypertension across the five districts in Lebanon. Selected findings from this paper have been presented at the American Society of Health-System Pharmacists (ASHP) conference (December 2015).

## Methods

### Study population and settings

This is a cross-sectional study, conducted between December 2014, and May 2015, on Lebanese adults aged 18 years and above. The participants were randomly enrolled from the five main districts of Lebanon to represent various geographic areas. These regions are the main governorates of Lebanon including; North, South, Beqaa, Mount Lebanon and the capital of Lebanon, Beirut. According to current national registries, the urban areas of Lebanon such as Mount Lebanon and Beirut are the most heavily populated, with about 75% of inhabitants living there. The remaining districts include both urban population in the main cities and rural communities. Over all districts, the Lebanese population is young with almost half of the population (47.1 percent) under 25 years of age and with life expectancy of 72 years. An approval for the study was received from the Institutional review board of the research council at School of Pharmacy at the Lebanese International University.

### Data collection

Multistage sampling was done by including several malls, street shops, universities and centers from each region starting with the capital city, Beirut, and followed over few weeks in other districts. An oral consent was provided from all participants to be interviewed and enrolled in the study. These participants were subjected to face-to face interviews to fill a questionnaire. Pregnant females and individuals who filled the questionnaire but did not have their blood pressure measured were excluded from the study. If any data was missing in the questionnaire, the subject was excluded from the study.

### Study questionnaire

A study questionnaire was presented to the participants by two registered pharmacists. It included participant’s demographics. Hypertension risk factors, including fat and salt intake, sedentary lifestyle, smoking and alcohol intake, dyslipidemia, diabetes mellitus, stroke, kidney disease, coronary artery disease and family history of hypertension. Diet was based on participant’s assumption of the amount of fat and salt intake per week. Sedentary life style definition was based on physical activity; participants were either classified as physically active or inactive^5^. To assess awareness, subjects were asked the following questions: Has a doctor ever said that you have high blood pressure or hypertension? Are you aware that it is a chronic disease and you shouldn’t stop your medication even if your BP is normal? Are you aware of its complications? For treatment assessment, subjects were asked whether they are taking any antihypertensive medications.

### Methods of measurement

Pharmacists used Omron blood pressure device (Omron 73237, Germany) to measure the participants’ blood pressure^6^. This is a calibrated automated digital blood pressure device. It is clinically proven to be accurate with a 2% rate of error. Subjects were asked to rest in a seated, upright position for 1-2 minutes, with back supported, legs uncrossed and arm extended at the same level of the heart. Participants with reported smoking, alcohol intake, or intense physical activity for the last 30 minutes were invited to rest for 30 minutes before measurement of blood pressure. Blood pressure was measured twice, each time in a different arm, the mean of these two was recorded. The participants’ weight in kilograms and height in meters were measured using.... The body mass index (BMI) was calculated as (weight (kg)/height (m)) x height (m).

### Variable definitions

Hypertension was defined as average measured blood pressure ≥140 mm Hg systolic and/ or 90 mm Hg diastolic, or self-reported use of medications for hypertension^7^. Participants were defined as aware of hypertension if classified as having hypertension and reported being informed about that by a medical provider. Participants were defined as treated if classified as having hypertension and reported that they were taking antihypertensive medications. Participants were defined as controlled if they were classified as having hypertension, receiving antihypertensive therapy and having a blood pressure measurement <140/90 mm Hg^7^.

### Statistical analysis

Descriptive analysis such as rate and proportion were used to estimate the prevalence, awareness, treatment and control of hypertension. Chi-square and crosstabs were used to compare the difference between variables. Logistic regression was used to assess the independent effects of individual factors. A ‘P’ value of less than 0.05 was considered to be significant. The Statistical Package for Social Science (SPSS) version 21.0 was used to analyze the data.

## Results

### Study population

This study recruited 1362 participants from the five districts of Lebanon: 105 from Beirut (7.7%), 922 from North (67.9%), 102 from South (7.5%), 85 from Mount Lebanon (6.2%) and 144 from (10.6%) Bekaa. Demographic information and lifestyle habits of the participants are shown in Table 1. Overall, among the participants 49.18% were males and 50.8% were females. The age of participants ranged between 18 years and 95 years with a mean age of 31 years, males had a mean age of 32.5 years ±15.3 and females 29.8 years ±13.3. Concerning location, the mean age of participants from North, Beirut, South, Bekaa and Mount Lebanon districts were 30.7 ± 14.4, 40.9 ± 15.55, 38.9 ± 17.33, 26.8 ± 10.27 and 21.9 ± 3.8 respectively. The mean BMI was 25 kg/m^2^. While 52.6% of the participants were smokers, 62.7% were physically inactive. 45.2% were either overweight or obese and 50.7% had a family history of hypertension.

**Table 1:** Baseline characteristics of participants.

	Men		Women		*P* value
	N	%	N	%	
**Age (years)**					
18-29	374	28.3	434	32.5	<0.05
30-39	94	7.04	93	6.97	
40-49	89	6.67	77	5.77	
50-59	47	3.52	50	3.75	
60-69	25	1.87	15	1.12	
>69	27	2.02	9	0.67	
Total	656	49.18	678	50.8	<0.05
** Regions**					
Beirut	46	3.4	59	4.3	0.3
North	451	33.2	471	34.7	
South	63	4.6	39	2.9	
Mount Lebanon	45	3.3	40	2.9	
Bekaa	58	4.3	86	6.3	
** BMI**					
Normal	243	21.54	308	27.39	0.05
Overweight	209	18.52	121	10.72	
Obese	102	9.04	77	6.83	
** Smoker**					
Yes	399	30.1	297	22.4	0.1
** Educational Level**					
No schooling	18	1.4	7	0.5	0.05
Primary School	90	6.9	88	6.7	
Secondary School	123	9.4	119	9.1	
University	410	31.2	458	34.9	
** Diet (fat/salt)**					
Low	117	8.9	126	9.6	0.9
Moderate	320	24.4	377	28.7	
High	202	15.4	172	13.1	
** Physical activity**					
Inactive	344	26.5	469	36.2	0.71
Active	286	22.1	197	15.3	
** Family history**					
Yes	305	23.0	368	27.7	0.05

**Table 2:** Percentage of prevalence, awareness treatment and control of hypertension in participants.

Characteristics	Men %				Women %			
	Hypertension	Aware	Treated	Controlled within treated	Hypertension	Aware	Treated	Controlled within treated
** Age (years)**								
18-29	32.6	10.8	2.5	0	10.8	7	4.5	4.8
30-39	38.3	20	8.6	5.9	17.2	26.7	20	9.5
40-49	44.9	37.5	22	17.6	35.5	26.1	21.7	14.3
50-59	59.6	36.7	32.3	29.4	62	54.8	54.8	42.9
60-69	51.7	64.7	47.1	17.6	73.3	54.5	54.5	23.8
>69	69.9	66.7	66.7	29.4	44.4	66.7	75	4.8
** Regions**								
Beirut	39.1	35.3	27.8	17.6	27.1	33.3	37.5	19
North	39.0	28.1	16.3	70.6	*20.4*	32.3	27.8	66.7
South	38.1	26.1	25	11.8	28.2	20	27.3	4.8
Mount Lebanon	35.6	18.8	6.3	0	10	66.7	33.3	4.8
Bekaa	37.9	4.5	4.5	0	15.1	7.7	7.7	4.8
** BMI**								
Normal	28	20.6	11.4	20	12	8.8	5.9	5.6
Overweight	41.1	26.1	18.2	60	24	42.9	39.3	44.4
Obese	58.8	33.3	19	20	44.2	38.2	38.2	50.0
** Smoker**								
Yes	40.4	21.9	11.3	47.1	23.2	38.1	34.4	61.9
** Educational Level**								
No schooling	2.8	3.2	0	5.3	27.5	2.7	2.9	4
Primary School	14	21	22.5	10.5	37.5	37.8	35.3	23.4
Secondary School	19.2	22.6	32.5	31.6	20.2	18.9	20.6	19.4
University	64	53.2	45	52.6	14.8	40.5	41.2	53.2
** Diet**								
Low	18.3	43.9	54.8	55	18.7	39.5	41.2	52.2
Moderate	50.5	39.4	38.1	40	55.9	47.4	52.9	43.5
High	31.6	16.7	7	5	25.5	13.2	5.9	4.3
** Physical Activity**								
Inactive	42.4	26	16.6	47.1	20.5	25.8	21.7	66.7
Active	109	70	46.6	53	57.1	103.5	104.8	33.3
** Family History**								
Yes	45.2	55.6	33.3	64.7	25.3	100	66.7	70

### Prevalence of hypertension

Among participants included in the study (Table 2), 399 were hypertensive (29.3%), 69 patients (65%) of those aware, were receiving treatment, and 38 (55%) participants from those treated were controlled. The age-standardized prevalence of hypertension was 29.2%. In the age category below 60 years, the prevalence of hypertension was higher in males whereas, in the age category 60–69, women had higher prevalence of hypertension. Moreover, male participants aged 69 years and above had a higher prevalence of hypertension (69.9%) compared to female participants (44.4%; *P* < 0.05).

### Risk factors

Smoking also played a role among hypertensives, where smokers had higher prevalence (59.3%) than nonsmokers (40.7%). For the education, 24% of participants with a university degree were hypertensive and 48% of participants who were not schooled were hypertensive (*P* < 0.05). Based on logistic regression, male gender had 2.4 times higher risk of suffering from hypertension than females. Obesity played an indispensable role in hypertension prevalence. The definition of obesity was based on the BMI; a BMI above 30 kg/m^2^ was considered obese. Prevalence increased significantly among obese males (58.8%) and females (44.2%) compared to males and females who have a normal body weight (28% and 12% respectively; *P* < 0.05). Moreover, obese people had 7.2 times higher risk of developing hypertension than people with normal weight. The increase in age also increase hypertension prevalence, as old people were 2.7 times more prone to suffer from hypertension than normal people.

### Awareness, treatment and control proportions

The percent of awareness about hypertension was higher in females (29.8%) than in males (25.9%; *P* = 0.2). The percentage of aware hypertensive participants receiving treatment were 74.5%. The rate of awareness was highest in Beirut area (34.4%) and lowest in Beqaa area (5.7%; *P* = 0.04). The percentage of aware hypertensives being treated was (74.5%), and a higher proportion of aware females (84.2%) were treated than aware males (56.9%; *P* < 0.05). From those treated, 54.4% were controlled. The control rate was higher in participants with low salt and fat diet (58.3%) than those with high salt and fat diet (20%; *P* = 0.16).

## Discussion

This study shows a high prevalence of hypertension among the Lebanese adult population with moderate awareness and treatment rates. The prevalence was evident in young male adults between 18 and 29 years. About half of the participants who were treated were controlled on their medication therapy. Low educational level, obesity and male gender played a significant role in increasing prevalence risk. Awareness rate was highest in Beirut, the capital of Lebanon. Treatment rate was higher among females, and control rate was higher in those on a low salt and low fat diet. The results of this study have valuable implications for cardiovascular health in Lebanese adults. About one third of Lebanese adults studied in our survey are hypertensive, and less than one third of those are aware. This indicates an urgent need for better follow-up in the Lebanese population, and to train health providers on issues of hypertension detection and surveillance.

On the other hand, about two-thirds of aware population received treatment, indicating that good access to healthcare will allow proper detection of the condition and hence the use of antihypertensive drugs when indicated. About half of the hypertensive treated patients achieved good control of blood pressure, indicating that management of hypertension by lifestyle measures and medications needs to be reinforced in the Lebanese community.

This study is among the few available in Lebanon about hypertension. In 2005 a study was conducted by Tohme et al., by administrating a questionnaire to 2125 adults aged equal or above 30 years old from all regions in Lebanon, resulted in a hypertension prevalence of 23%^8^. The prevalence from that study may not be conclusive however, since high blood pressure was reported by participants as a yes/no question and through oral interviews, but without blood pressure measurement.

Another study was conducted in January 2015 by Matar et al. and showed a hypertension prevalence of 36.9%^4^. Lower percentages were reported in our study, this may be explained by the older sampled population by Matar et al. (above 21 years old) versus our study (above 18 years old). Another factor that may have contributed to such a difference is that our study included some university students with fewer risk factors for hypertension. Our study found rate of awareness of hypertension to be 26.5%, a lower rate than that reported by Matar et al. as 53%, and this may be explained by the fact that their population was mostly based on Beirut and Mount Lebanon, which are urban areas with modernized lifestyle and probably better awareness.

Sixty-five percent of our participants with hypertension were receiving blood pressure medications, versus 48.9% in the previous study by Matar et al., while the control rates of treated hypertensive patients were similar (55% in our study and 54.2% in Matar et al. study). These differences and similarities indicate heterogeneity of the conducted studies and possible differences among studied populations, sampling methods, study settings, and time period when the study was conducted. These findings warrant better follow-up of the status of hypertension treatment and control among Lebanese adults, and perhaps stress the need for more comprehensive, consolidated, and probably long-term studies.

A systematic review about hypertension prevalence, awareness, and control in Arab countries has shown that the prevalence of hypertension increased with age and occurred more frequently in Arab women. The overall estimated prevalence of hypertension in the Middle East was 29.5%^3^. This conforms to results of our study and indicates that Lebanon has an average prevalence of hypertension compared to neighboring countries.

Awareness of hypertension was 46% and varied from 18% (Jordan) to 79.8% (Syria)^3^. A national survey about hypertension prevalence, awareness, treatment and control, and its associated factors in Jordan showed that the prevalence of hypertension was 32.3% in adults aged 25 years or more^9^. The rate of awareness among those hypertensive patients was 56.1%^9^. Another national survey about hypertension in the Kingdom of Saudi Arabia (KSA) in 2013 found that 15.2% were hypertensive, noting that KSA has a young population with 81% of the population under the age of 40^10^.

A study in Tunisia on elderly patients (age ≥ 65 years) resulted in a prevalence of 52% among studied individuals^11^. Overall, according to our findings, Lebanon’s rates of hypertension prevalence are not alarming compared to the region, and are better than some countries. The rates of awareness are lower than some countries like Jordan, probably due to better socioeconomic variables, but higher than some countries like Syria, after the crisis of the civil war.

Our study found a clear correlation between awareness, treatment and control of hypertension in a manner of logical sequence. Being aware of having hypertension led to higher chances of being treated and therefore being controlled. The prevalence of hypertension was higher in males than in females which is parallel to the latest study in Lebanon^4^. The prevalence of hypertension in 2005 was lower according to a study by Tohme et al. and was 23.1%^8^. Hypertension prevalence was higher among older age groups. This finding is consistent with the fact that hypertension prevalence increase with age^9^. Comparing Beirut, being the capital and an urban district, to Beqaa, being a rural district, prevalence of hypertension was higher in Beirut. These results were reported elsewhere; a study by Chaturvedi et al. showed an association between increase blood pressure and urbanization^12^.

Furthermore, male gender, family history of hypertension, obesity, and family history of hypertension were significant risk factors for hypertension. Among hypertensive participants, more than half were smokers^13,14^. These results are consistent with prospective study done on women showing that heavy smoking results in persistent rise in blood pressure^15^. Moreover, overweight and obesity play a direct role in increasing the prevalence of hypertension compared to normal and underweight participants^16^. This study was the latest in Lebanon to assess the relation between risk factors and hypertension in the country. In a study from January 2015, significant risk factors for hypertension prevalence were age, male sex, being widowed, smoking, obesity, diabetes mellitus and dyslipidemia^4^. In a study from 2005, the significant risk factors for hypertension prevalence were age, low education level and retired individuals^8^.

In neither males nor females could we determine a clear correlation between educational level and awareness about hypertension. On the other hand, awareness was highest in the two urban districts: Beirut and Mount Lebanon. This sounds logical, but conclusions are hard to draw due to the small sample size compared to other regions.

Awareness among older participants was highest. This can be explained by the frequent visits to medical centers by older participants due to their comorbidities and assessment by healthcare providers, thus, informing them about their high blood pressure.

Treatment and control of hypertension were two strongly related factors according to our findings, where 55% of participants controlled were treated. The widespread lack of hypertension control may be due to high costs of accessing medical care, inadequate pharmacological prescription, or poor compliance, or poor lifestyle^17,18^.

## Strengths and limitations

This study had limitations. One was measuring blood pressure in different settings where participants were selected, such as street shops, malls, universities and others. This method of cross-sectional sampling results in participants that do not necessarily represent the general population. Multistage sampling from home units would have been better for data collection because they may be more representative. Our data collection also avoided to choose locations that usually include a bulk of hypertensive patients like hospitals. The percentage of participants in the 18-29 age group was highest. Although some hypertension was detected in this age category, overall the prevalence of hypertension may have decreased due to this sampling issue, indicating the need for an older age category.

Moreover, small sample size and recall bias may have occurred with some participants while answering the questionnaire. Although the sample size was comparable between Beirut and Bekaa, the difference in prevalence of hypertension may be explained by taking a larger sample size from university students in Bekaa. Blood pressure measurement was done on a single encounter and this differs from current guidelines of diagnosis of a high blood pressure being done on two different encounters. This may affect the overall sensitivity of our measurements. Also, blood pressure was measured twice for each participant instead of three times as recommended in some guidelines.

The strengths of this study lie in the recruitment of participants from rigorous geographical areas, different age groups, variability in lifestyle factors and health status. Moreover, interviews and blood pressure measurements were done by trained pharmacists using standardized BP measurement device. Another advantage is the assessment of several risk factors in the study and the assessment of relation between socioeconomic and other backgrounds to hypertension.

## Conclusion

It is evident that hypertension is a common public health problem in Lebanon with complex etiologies, but is not well acknowledged leading to its poor control. This is the most updated cross-sectional study carried out in Lebanon that correlates hypertension with its risk factors. The results show important gaps in the management of hypertension in this country and that substantial improvement in hypertension education, treatment, and control is needed. The highest concern is that about half of individuals with known hypertension are not controlled. Therefore, the healthcare community is responsible to improve awareness, treatment, and control of hypertension by thorough detection and treatment methods, aiming to decrease the overall prevalence and mortality associated with hypertension in Lebanese adults.

## Competing interests

None declared.

## Funding

The funding of the study, which included blood pressure monitors, body mass scales and transportation were on the expense of the pharmacists enrolling the study; no additional funding was provided.

## Patient consent

Patient consent was obtained verbally from Lebanese adults who accepted to participate in the study.

## Ethics approval

The study was approved by the Institutional Review Board (IRB) at Lebanese International University School of Pharmacy. The population only included Lebanese adults who accepted to participate in the study after an oral informed consent was provided.

## Data sharing statement

Extra data is available by emailing the corresponding author.
